# Epimutation and Cancer: Carcinogenesis Viewed as Error-Prone Inheritance of Epigenetic Information

**DOI:** 10.1155/2018/2645095

**Published:** 2018-06-03

**Authors:** Patrick A. Riley

**Affiliations:** Totteridge Institute for Advanced Studies, The Grange, Grange Avenue, London N20 8AB, UK

## Abstract

The epimutation concept, that is, malignancy is a result of deranged patterns of gene expression due to defective epigenetic control, proposes that in the majority of adult cancers the primary (initiating) lesion adversely affects the mechanism of vertical transmission of the epigenetic pattern existing in the stem cells of differentiated tissue. Such an error-prone mechanism will result in deviant gene expression capable of accumulation at each mitosis of the affected stem cell clone. It is argued that a proportion of these proliferation products will express combinations of genes which endow them with malignant properties, such as the ability to transgress tissue boundaries and migrate to distant locations. Since the likelihood of this occurrence is dependent on the proliferation of cells manifesting the defective epigenetic transmission, the theory predicts that cancer incidence will be strongly influenced by factors regulating the turnover rate of the stem cells of the tissue in question. Evidence relating to this stipulation is examined. In addition, it would be anticipated on the basis of the selection of genes involved that the susceptibility to malignant transformation will vary according to the tissue of origin and this is also discussed.

## 1. Introduction

Recently there has been considerable interest in the role of epigenetic mechanisms in cancer [1–3] and it has been proposed that deranged epigenetic regulation is the crucial lesion of carcinogenesis. Such a scenario would account for the many deviant characteristics exhibited by malignant and premalignant cells [4]. These include multiple derangements of structure and metabolism and the emergence of cellular properties associated with different tissues and with embryonic stages of development, especially the metastatic ability to transgress tissue barriers and migrate to distant sites which is the defining characteristic of malignant neoplasms.

The cardinal abnormality exhibited by the majority of adult cancers is chromosomal instability (CIN) with widespread alterations in gene expression [5] accompanied by a range of diagnostic cytological aberrations [6]. Thus, the fundamental lesion at the root of this pathological process must be one that causes a general disturbance of the chromatin pattern and it has been proposed that this results from a failure to preserve the epigenetic markers during DNA replication [7]. According to this proposal the primary (initiating) lesion of carcinogenesis is the acquisition of one or more mutations that result, during stem cell mitosis, in defective vertical transmission of the epigenetic pattern characteristic of the differentiated tissue. If the development of this defect was equally likely for each tissue it might be anticipated that the age-specific incidence of all cancers would be similar, but the statistical data show that there are substantial tissue-specific differences [8] and these require explanation.

## 2. Differences in Cancer Incidence in Different Tissues

Some of the relevant factors involve obvious differences such as the number of susceptible cells and the degree of mutagenic exposure. For each tissue the probability of the initiation phase taking place is influenced by the size of the population, the number of genes that have to be mutated to bring about the defect, the exposure to mutagenic events, and the elimination or repair efficiency of the relevant stem cells, i.e., stem cell numbers, the number of crucial genes, and their effective mutation rate [9, 10]. Differences in the mutation rate and/or exposure to mutagens have been proposed to account for the observed variation in cancer incidence in different tissues and these considerations have received much detailed attention in the extensive extant literature and are not addressed in any detail in this brief review. However the relative size and turnover of the stem cell population at risk in a tissue are obviously a determining factor.

An important question with regard to the epigenetic theory of carcinogenesis concerns the problem of which genes are necessary and sufficient to bring about the defective copying of the epigenetic pattern and whether the same genes are involved in all cases. It can be argued that in developmental neoplasms the underlying problem is the failure of evocation of some gene silencing mechanism involved in differentiation, and in these cases reversal is possible [11]. In adult malignancies there are many genes implicated in epigenetic copying that could be affected [12–16] and relatively little is known about the control of homologous gene silencing and the effect of ploidy. Also, the involvement of a ratifying system has been suggested, such as the p53, related DNA editing machinery [17–20]. At present the impact of these matters remains unresolved.

However, assuming that the initiation stage has been accomplished, the factors implicated in the secondary carcinogenic events, i.e., the failure of fidelity of epigenetic copying resulting from the initiating lesion, have hitherto received relatively little discussion. In essence the likelihood of the acquisition of epigenetic errors that result in malignancy will depend on two criteria: (1) the rate of stem cell proliferation and (2) the ease of activation of the genes that determine the metastatic phenotype. In the absence of clear evidence of which genes are involved in the processes that result in the manifestation of the malignant phenotype, the second factor is difficult to assess. Possibly, if reactivation of the most recently silenced genes occurs more readily, it might be speculated, for example, that migratory properties would be more likely to be expressed in melanocytes, thus accounting for the earlier age-specific incidence of melanoma [8]. An interesting correlation between the extent of DNA methylation and the age-specific risk of malignancy has been demonstrated [21] which may indicate that cell classes with a higher proportion of silenced genes are more susceptible to malignant transformation.

## 3. Significance of the Stem Cell Proliferation Rate

From the theoretical point of view, the proposed central role of stem cell proliferation in bringing about the epigenetic errors that lead to the malignant phenotype makes a number of testable predictions, several of which are known to be the case. For example, it follows that malignant tumours are not found in nonproliferating tissues such as the central nervous system and rarely occur in slowly proliferating cells such as striated muscle. Moreover, since the greatest turnover occurs in epithelia, it accounts for the high proportion of epithelial cancers. The central significance of stem cell proliferation has been emphasised by the statistical association between cancer risk and the total number of stem cell divisions in different tissues as shown by Tomasetti and Vogelstein [22] and the epigenetic model is compatible with the observed age-specific cancer incidence in a number of specific cases where the relevant data are available [23]. The importance of the stem cell proliferation rate also accounts for the decreased cancer incidence rate with increasing age. This phenomenon has been analysed by Pompei and Wilson [24] and shown to correspond with a linear age-specific reduction. Another interesting example of the effect of the rate of stem cell proliferation and cancer incidence is the relatively low incidence of melanoma in blacks which is inversely proportional to the degree of melanisation and has been ascribed to the diminished stem cell turnover in regions where melanocyte proliferation is inhibited by loss of cytoplasmic volume through melanosome donation to adjacent keratocytes [25].

Also consistent with the epigenetic model is the evidence of increased risk of malignancy associated with factors increasing the proliferation rate of tissues. This includes the stimulatory effects of chronic inflammation and specific growth factors such as hormones, e.g., the increased breast cancer risk associated with hormone replacement therapy [26]. At the other end of the spectrum is the explanation of the sometimes-observed prolonged induction periods by nonproliferation of transformed cells.

The influence of the stem cell proliferation rate on the acquisition of malignant characteristics importantly predicts that initiated cells can be prevented from becoming malignant by suppression of proliferation. This phenomenon has been described in the case of hepatocytes experimentally initiated by aflatoxin [27–30].

## 4. Preventative and Therapeutic Prospects

In brief, the preventative possibilities presented by the epimutation model of cancer concern the identification of mutant genes responsible for the error-prone epigenetic copying and their repair or selective elimination of cells bearing these mutations. In this respect the scenario differs from the conventional theories of carcinogenesis only in focusing on genes implicated in the epigenetic copying mechanism. It is a moot point which genes might be most significant in this respect but possible candidates include genes involved in regulating the activities of DNA methylases, histone deacetylases, p53 and related processes involved in apoptosis, and other possible gatekeeping and editing functions. For example, polycomb group proteins such as EZH2 the H3K29 methylase UTX and components of the chromatin remodelling complex such as SWI/SNF are modified in several cancers [31] and there is evidence that certain drugs targeting epigenetic mechanisms show clinical promise [32] but the picture is complicated by the fact that once epigenetic error is introduced, the genomic interactions will result in abnormal combinations of hypo- and hypermethylation [33] so that the outcome of epigenetic interference cannot be easily predicted and may differ according to the affected tissue or the individual [34].

Assuming that malignant behaviour in all cases is derived from a specific genetic composition, a promising approach might lie in the identification of the genes giving rise to the crucial malignant properties. This would potentially enable the design of agents causing epigenetic suppression of these malignant genes or drugs to selectively eliminate cells expressing those genes. At present, the nature of such crucial genetic targets is not clear and, moreover, it seems probable that the route to malignancy varies according to the tissue of origin. For the present it appears that the most accessible target is the evidence that the carcinogenic risk is a function of the stem cell proliferation rate of the tissue in question. Hence, if a tissue has undergone carcinogenic induction, any means that diminishes the rate of stem cell proliferation will suppress the emergence of a malignant variant clone and, of course, many of the currently effective chemotherapeutic agents target the proliferation rate of the affected tissue.

## 5. Conclusions

The epigenetic theory of carcinogenesis proposes that the acquisition of the malignant phenotype results from error-prone copying of the epigenetic pattern when tissue stem cells divide. This failure of fidelity of epigenetic transmission is due to initiating mutations affecting the set of crucial genes involved in the normally stable copying of the epigenetic pattern. The manifestation of error-prone epigenetic copying is the generation of clones showing a diversifying range of abnormalities including structural and functional anomalies, abnormal mitoses, alterations of ploidy, and the occurrence of bizarre cytological features. It is argued that these anomalies give rise to cells some of which exhibit malignant characteristics and are able to transgress tissue barriers and spread to distant sites.

Whilst it may be impossible to avoid the initiating mutagenesis, in principle cancer could be prevented if cells bearing the initiating lesion(s) could be identified and the faulty epigenetic process corrected or the affected cells eliminated. This eventuality remains a hope for the future.

However, in view of the important role of mitosis in enabling the perpetuation of epigenetic errors, ensuring the minimum turnover rate of tissue stem cells seems to offer a basis for a general cancer prevention strategy.
